# P02-032 - CAPS: a novel mutation and an unusual phenotype

**DOI:** 10.1186/1546-0096-11-S1-A139

**Published:** 2013-11-08

**Authors:** A Insalaco, PS Buonuomo, C Bracaglia, M Pardeo, I Ceccherini, R Nicolai, F De Benedetti

**Affiliations:** 1 Division of Rheumatology, Department of Pediatric Medicine, Ospedale Pediatrico Bambino Gesù, Rome, Italy; 2 Molecular Genetic Laboratory, Istituto Giannina Gaslini, Genova, Italy

## Introduction

Cryopyrin-associated periodic syndrome (CAPS) is an autoinflammatory syndrome caused by heterozygous mutations of CIAS1/NLRP3 gene. Affected patients may present with three different phenotypes: familial cold autoinflammatory syndrome (FCAS), Muckle-Wells syndrome and CINCA syndrome. Common symptoms include sporadic or cold-induced non pruritic urticarial rash and fever. Severe cases suffer from deafness, meningitis, articular contracture and secondary amyloidosis.

## Case Report

We describe a 13-year-old female who complained, starting at 12 years of age, of recurrent episodes of high fever, pericarditis, arthralgia, arthritis of the knees, abdominal pain. These episodes were associated with marked increase in inflammatory markers and in serum amyloid level (5 episodes in the first 5 months of disease). Symptoms were poorly responsive to NSAIDs and colchicine but responded to steroid therapy. Molecular analysis of the MEFV, TNFR and MVK genes did not show any pathogenic mutations. In the subsequent months she developed recurrent (up to daily) episodes of chest pain, skin rash and swelling of the subcutaneous tissue of limbs, trunk, joints and lips, in the absence of fever, with spontaneous resolution.

Molecular analysis of the CIAS1 gene revealed the presence of a c.1105C>A mutation in the heterozygous state, that predicts a L369M amino acid substitution. To the best of our knowledge this variant has never been reported. One hundred chromosomes were examined and the variant was not found. In order to verify the potential pathogenic effects of the L369M amino acid substitution, daily therapy with anakinra (2 mg/kg/day) was started with rapid disappearance of clinical symptoms and normalization of CRP levels in 24 hours. Since the pathogenic significance of the mutation observed is not known, prediction of the effect of this mutation on the protein function has been attempted, in silico, by subjecting the p.L369M substitution to the Variant Effect Predictor tool. The mutation was predicted to significantly affect protein structure (scoring as “dangerous” and “deleterious”).

## Discussion

The fast response to IL-1 inhibition suggests that the disease of this patient is driven by IL-1 and support the conclusion that this novel mutation is pathogenic and may be associated with a new CAPS phenotype.

## Disclosure of interest

None declared
